# Usnic Acid-Loaded Magnetite Nanoparticles—A Comparative Study between Synthesis Methods

**DOI:** 10.3390/molecules28135198

**Published:** 2023-07-04

**Authors:** Cristina Chircov, Alexandra Cătălina Bîrcă, Lorena Alexandra Dănciulescu, Ionela Andreea Neacșu, Ovidiu-Cristian Oprea, Roxana-Doina Trușcă, Ecaterina Andronescu

**Affiliations:** 1 Department of Science and Engineering of Oxide Materials and Nanomaterials, University Politehnica of Bucharest, 011061 Bucharest, Romania; cristina.chircov@yahoo.com (C.C.); ada_birca@yahoo.com (A.C.B.); truscaroxana@yahoo.com (R.-D.T.); ecaterina.andronescu@upb.ro (E.A.); 2 National Research Center for Micro and Nanomaterials, University Politehnica of Bucharest, 060042 Bucharest, Romania; 3 Faculty of Medical Engineering, University Politehnica of Bucharest, 060042 Bucharest, Romania; lorenadanciulescu@gmail.com; 4 Department of Inorganic Chemistry, Physical Chemistry and Electrochemistry, University Politehnica of Bucharest, 011061 Bucharest, Romania; 5 Academy of Romanian Scientists, 54 Spl. Independentei, 050045 Bucharest, Romania

**Keywords:** magnetite nanoparticles, usnic acid, anticancer, co-precipitation, microwave-assisted hydrothermal synthesis

## Abstract

Since cancer is a continuously increasing concern for the general population, more efficient treatment alternatives ought to be developed. In this regard, a promising direction is represented by the use of magnetite nanoparticles (MNPs) to act both as a nanocarrier for the targeted release of antitumoral drugs and as hyperthermia agents. Thus, the present study focused on improving the control upon the outcome properties of MNPs by using two synthesis methods, namely the co-precipitation and microwave-assisted hydrothermal method, for the incorporation of usnic acid (UA), a natural lichen-derived metabolite with proven anticancer activity. The obtained UA-loaded MNPs were thoroughly characterized regarding their morpho-structural and physicochemical properties through X-ray diffraction (XRD), Fourier-transform infrared spectroscopy (FT-IR), dynamic light scattering (DLS) and zeta potential, scanning electron microscopy (SEM), and vibrating sample magnetometry (VSM). Results demonstrated the formation of magnetite as the unique mineralogical phase through both types of synthesis, with increased uniformity regarding the drug loading efficiency, size, stability, and magnetic properties obtained through the microwave-assisted hydrothermal method. Furthermore, the cytotoxicity of the nanostructures against the HEK 293T cell line was investigated through the XTT assay, which further proved their potential for anticancer treatment applications.

## 1. Introduction

The process of obtaining nanostructured drug delivery systems is under continuous expansion, with novel nanocarrier types and synthesis methods being constantly developed and/or improved [1,2]. Among them, magnetite nanoparticles (MNPs) are one of the most intensively studied types of nanomaterials owing to their unique magnetic properties that allow for their application against a variety of diseases, such as cancer and microbial infections [3,4,5].

The potential of MNPs is further enhanced by the possibility to apply them in hyperthermia treatment. Hyperthermia represents an adjuvant anticancer therapy based on the local elevation of temperature above physiological levels, i.e., 40–43 °C, in order to destroy proteins and cell structures. In cancer therapy, over four decades of research have shown that hyperthermia is able to damage or even kill cancer cells, thus causing the shrinking of tumors. In this manner, hyperthermia is currently being applied in combination with chemotherapy or radiotherapy for treating numerous tumor types, such as recurrent breast cancer, cervical carcinoma, melanoma, soft tissue sarcoma, and bladder cancer [6,7]. When using MNPs, temperature elevation is achieved through the application of an alternating external magnetic field that causes the MNPs to generate heat within the surrounding tissue [8,9]. In this context, MNPs represent a promising alternative in cancer therapy as they can concomitantly act as drug delivery carriers for the targeted administration of chemotherapeutics and as hyperthermia agents for heat generation.

Since conventional chemotherapeutics are known to cause serious side effects when administered, current research is focusing on the use of alternative, natural biocompounds that could destroy tumor cells while minimizing the associated risks [10,11]. Usnic acid (UA) is a natural dibenzofuran derivative present in lichens, which are organisms resulting from the symbiosis between a cyanobacterium or an alga and a fungus [12,13]. Its first documented use for the treatment of malaria, snakebite, wounds, and pulmonary tuberculosis dates back to the first century B.C. [14,15]. Due to the three-ketone groups, the furan ring that bonds the aromatic rings, as well as the intramolecular hydrogen bridges within its chemical structure, UA is a hydrophobic compound with a water solubility of less than 10 mg/100 mL at room temperature [15,16]. Hence, its administration within the human body is considerably limited, thus requiring the use of a nanocarrier for its successful delivery.

In this manner, the present study focused on the development of UA-loaded MNPs for potential applications in cancer therapy. Furthermore, considering the increasing need for standardization in nanomaterial science, the drug delivery systems were obtained through two synthesis methods, namely co-precipitation and microwave-assisted hydrothermal method, in order to achieve a higher control over the outcome properties of the nanostructures. Thus, the obtained UA-loaded MNPs were extensively characterized in terms of morpho-structural and physicochemical properties to investigate the advantages of the applied synthesis methods, as well as regarding their cytotoxicity against the HEK 293T cancer cell line for further cancer treatment applications.

## 2. Results

Within the present study, a series of UA-loaded MNPs were synthesized through two methods, namely, co-precipitation and microwave-assisted hydrothermal synthesis, in order to compare the outcome properties of the drug delivery systems (Table 1). In this manner, the obtained nanoparticles were characterized through XRD, FT-IR, DLS and zeta potential, SEM, and VSM analyses. Furthermore, the anticancer potential of the nanostructured systems was assessed through the XTT assay using the HEK 293T cell line.

**Figure 1:**
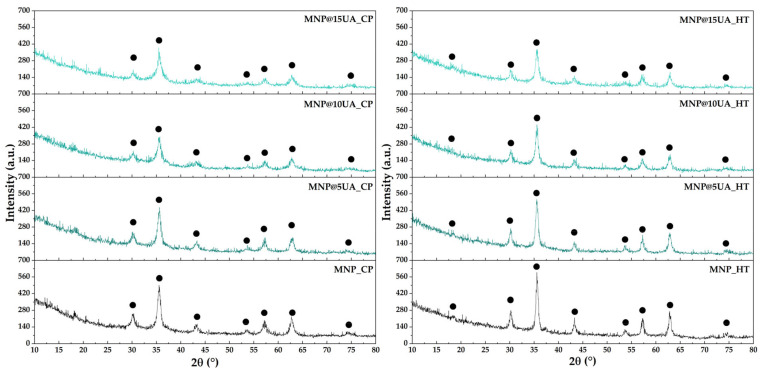
Diffractograms for the pristine and UA-loaded MNPs obtained through co-precipitation (**left**) and microwave-assisted hydrothermal method (**right**) (●—magnetite).

The XRD analysis was employed for assessing the mineralogical phases present within the samples, while the Rietveld refinement allowed for the determination of the unit cell parameters and the average crystallite size. As the diffractograms show (Figure 1), magnetite in the Fd–3m cubic crystal system is the unique crystalline phase within all samples (according to JCPDS 01-084-2782 [17,18]). Therefore, it can be concluded that despite the high pressure and temperature conditions for the reaction, microwave-assisted hydrothermal synthesis does not lead to the formation of secondary phases. Additionally, this method allows for the formation of nanoparticles with increased crystallinity, as the intensity of the diffracted radiation is higher than in the case of MNPs obtained through co-precipitation. The Rietveld fitting (Table 1) showed slight decreases in the unit cell and, consequently, in the average crystallite size inversely proportional to the UA concentration. Furthermore, results demonstrate an increase in the average crystallite size for the samples obtained through the microwave-assisted hydrothermal method, which could be explained by the growth of the nucleation centers due to the thermal and pressure treatment.

**Figure 2:**
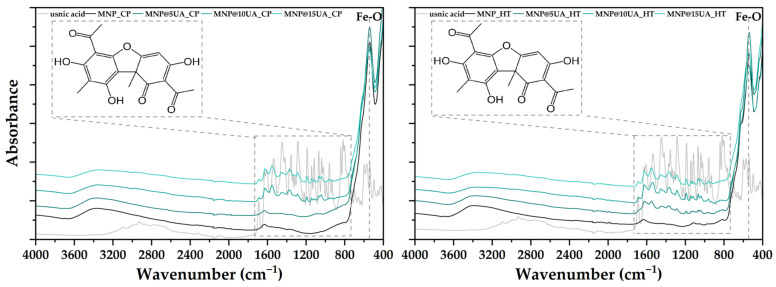
FT-IR spectra for the pristine and UA-loaded MNPs obtained through co-precipitation (**left**) and microwave-assisted hydrothermal method (**right**) (marked square—UA fingerprint).

Subsequently, the FT-IR analysis allowed for the determination of the chemical bonds present within the samples (Figure 2). As can be seen, the absorbance maximum at ~540 cm^−1^ specific for the Fe–O bond appears in all samples, with its intensity decreasing with increasing UA concentration. Furthermore, there are no shifts of the maximum after the addition of UA, which means that there are no changes within the structure of the Fe–O bond. Thus, it would be expected that the UA molecules were bound to the surface of the MNPs through hydrogen bonds, specifically between the hydrogen atom within the hydroxyl groups present onto the surface of magnetite and the oxygen atoms present within the UA molecule. The presence of UA is also demonstrated through the absorption bands between 800 and 1800 cm^−1^, characteristic for the aromatic or methyl group-associated C–H bending, aliphatic C=O stretching, phenolic and alcoholic O–H bending, and C–O stretching from an alkyl aryl ether, which are part of the compound fingerprint [19]. Considering the intensity of the absorbance peaks, it is safe to assume that the microwave-assisted hydrothermal method provides an improved loading capacity compared to the co-precipitation technique, especially in the case of the 5% concentration.

**Figure 3:**
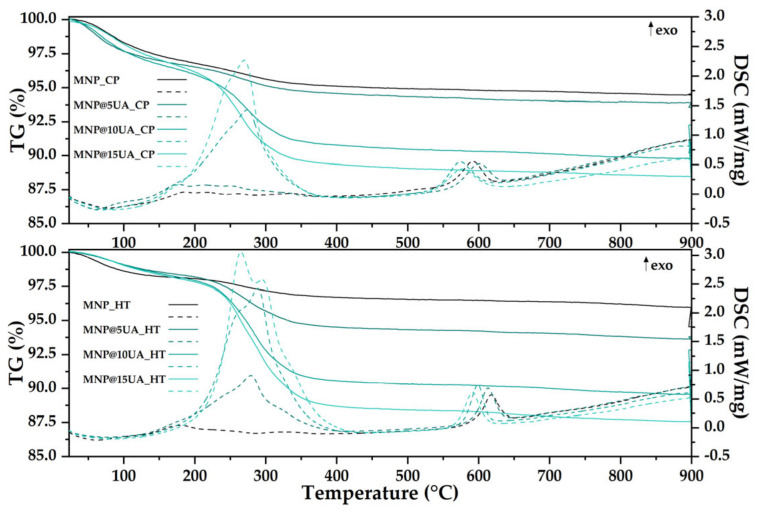
TG-DSC curves for the pristine and UA-loaded MNPs obtained through the co-precipitation (**up**) and microwave-assisted hydrothermal method (**down**).

Furthermore, the amount of loaded UA was determined through TG-DSC analysis (Figure 3), which can be divided into three temperature intervals. In the first interval, up to 200 °C, the samples are presenting a mass loss of ~3.20–4.10%, accompanied by an endothermic process with minimum around ~61.9–86.4 °C. This can be assigned to a dehydration process, with the surface-bound water molecules being eliminated, as indicated by the FTIR spectra of the evolved gases [20]. In the second interval, between 200–400 °C, the samples exhibit a mass loss of ~1.70–6.79%, assigned to the partial degradation of the organics (UA) loaded on the surface of the nanoparticles. In the same interval, the Fe(II) ion is oxidized to Fe(III), the magnetite being transformed to γ-Fe_2_O_3_ (maghemite) [21]. The process is composed of multiple reactions, backbone fragmentation and oxidation, as indicated by the asymmetry of the effects from the DSC curve. The FTIR spectra of the evolved gases indicates the presence of water and CO_2_ in this temperature interval, pointing out to the oxidation of the capping substances. In the third temperature interval, the carbonaceous residual mass is burned away. The characteristic exothermic effect from ~574–601 °C is assigned to the physical transformation of maghemite to hematite (γ-Fe_2_O_3_ to α-Fe_2_O_3_) [22]. The residual mass of the samples and the estimated load are presented in Table 2.

As it can be seen, the microwave-assisted hydrothermal method ensures a higher UA loading capacity, thus confirming the previous FT-IR observations. Moreover, the MNP_5UA_CP sample appears to have loaded an unsignificant amount of UA, which was also previously seen on the FT-IR spectra.

**Figure 4:**
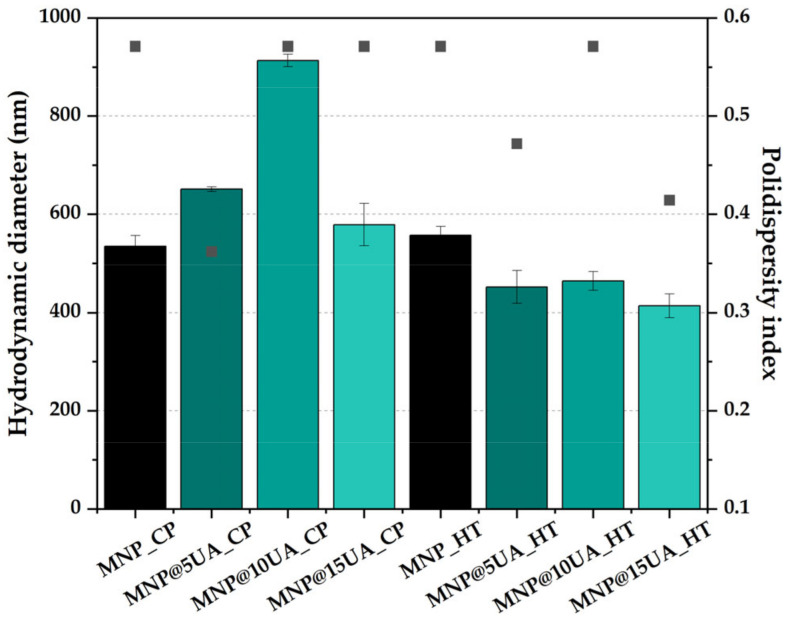
Hydrodynamic diameter (shown as columns) and polydispersity index (shown as points) values for the pristine and UA-loaded MNPs (expressed as mean ± SD, n = 3) measured in PBS 1×.

In regard to the hydrodynamic diameters (Figure 4), two types of behaviors can be observed for the two types of synthesis methods. While the hydrodynamic diameter values are similar for the pristine MNPs, the addition of UA leads to an increase for the systems obtained through co-precipitation and a decrease for the systems synthesized through the microwave-assisted hydrothermal method. These results are in accordance with the mass losses registered in the 20–200 °C temperature interval, which showed that the systems obtained through the co-precipitation method are characterized by a higher degree of surface-bound water molecules. The hydrodynamic diameter distribution for each sample can be seen in Figures S1–S8 (Supplementary Material). It can be observed that in the case of pristine MNPs, the microwave-assisted hydrothermal method leads to narrower size distributions.

**Figure 5:**
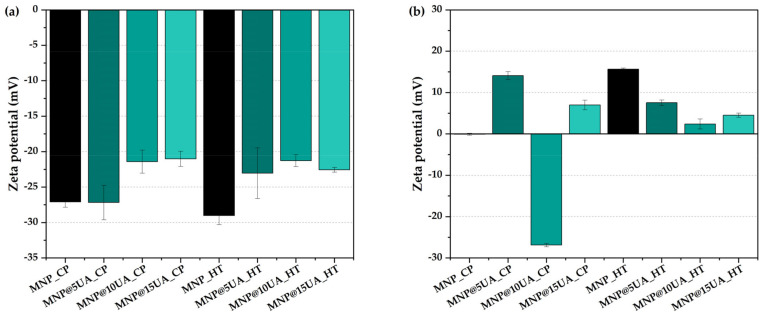
Zeta potential values for the pristine and UA-loaded MNPs measured in PBS 1× (**a**) and deionized water (**b**) (expressed as mean ± SD, n = 3).

Furthermore, increasing the UA concentration leads to lower zeta potential values (Figure 5a) measured in PBS, which is in accordance with previously published literature [23] and could be justified by the hydrophobicity of UA [24]. However, the MNP@5UA_CP sample shows a similar zeta potential value as the pristine sample, which is explained by the significantly reduced loading, i.e., 0.58%. It is worth mentioning that if samples are dispersed in PBS, the pH and ionic strength are controlled, although the surface of the MNPs is modified due to specific adsorption of phosphate ions [25]. In this case, MNPs are negatively charged due to the phosphate ions adsorbed onto the surface of the MNPs. However, as the phosphate adsorption governs the charge state of the nanoparticles, the UA load cannot be interpreted with zeta potential values. By contrast, when measuring the zeta potential in deionized water (Figure 5b), the particles appear to be more aggregated due to the lack of salts present within the solvent and the pH value close to the isoelectric point of the nanoparticles.

**Figure 6:**
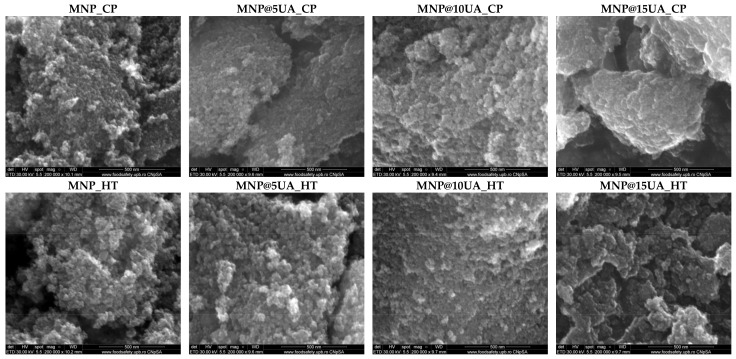
SEM images acquired for the pristine and UA-loaded MNPs.

**Figure 7:**
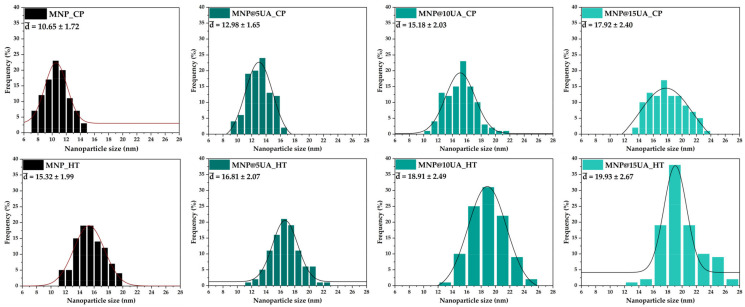
Size distributions and average nanoparticle size for the pristine and UA-loaded MNPs.

The SEM images were acquired for investigating the morpho-structural features of the nanostructured systems (Figure 6). All samples are characterized by a quasispherical shape and an increased agglomeration tendency owing to the large surface area of the nanoparticles. As the histograms show (Figure 7), the microwave-assisted hydrothermal method leads to the formation of larger nanoparticles with more uniform size distributions, which further confirms the advantage of controlling the outcome properties of the nanoparticles. Furthermore, increasing the amount of UA added is proportional to the nanoparticle size in both types of synthesis methods.

**Figure 8:**
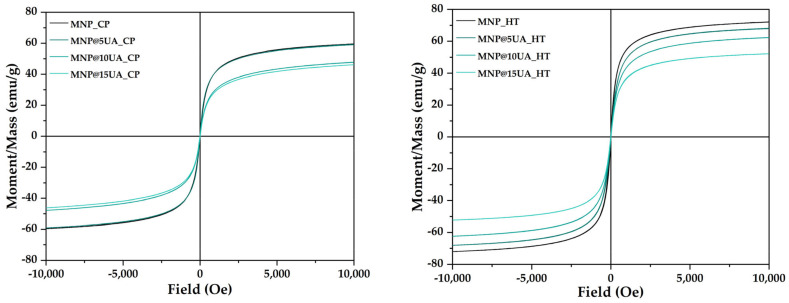
Field-dependent magnetization curves for the pristine and UA-loaded MNPs obtained through the co-precipitation (**left**) and microwave-assisted hydrothermal method (**right**).

The VSM analysis was employed for assessing the magnetic properties of the nanostructured drug delivery systems (Figure 8). In all samples, the magnetization curves present no hysteresis, thus proving the superparamagnetic behavior of all types of nanostructured systems [26,27,28,29,30]. The saturation magnetization values decrease with the increase of the UA content, the highest values being registered for the pristine MNPs in both cases. The hydrothermal method results in the formation of nanoparticles with higher saturation magnetizations, probably due to larger nanoparticle sizes, as shown in the SEM images. The saturation magnetization, remanence magnetization, and coercivity field values of the pristine and UA-loaded MNPs are shown in Table 3.

**Figure 9:**
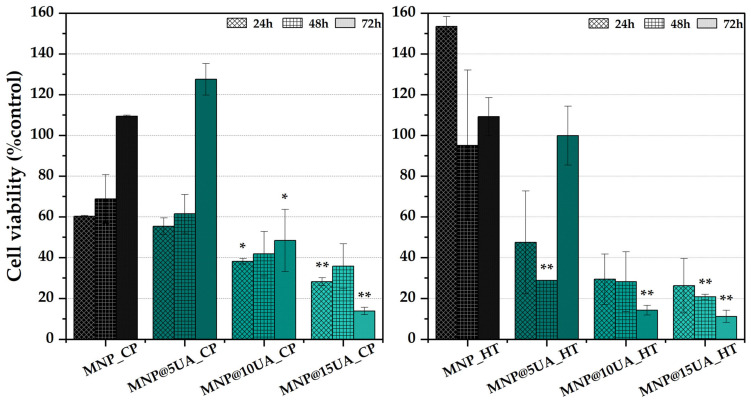
Cell viability results for the pristine and UA-loaded MNPs obtained through co-precipitation (**left**) and microwave-assisted hydrothermal method (**right**); level of statistical significance—* *p* < 0.05 and ** *p* < 0.01 to the control.

The antitumoral potential of the developed nanostructures was investigated through the XTT assay using the HEK 293T cell line (Figure 9). Considering the pristine MNPs, it can be seen that the cell viability is higher for the nanoparticles obtained through the microwave-assisted hydrothermal method at all three time-points, possibly due to their increased size which delays the internalization of the nanoparticles within the cancer cells. Furthermore, in regard to the UA-loaded samples, they are more cytotoxic towards the HEK 293T cells due to an improved drug loading efficiency, which further confirms previous results from FT-IR and TG-DSC. Additionally, the cell viability decreases in time, thus proving the prolonged release of UA. While in the case of the co-precipitation method where the most effective results were registered for the 15% concentration, the 10% concentration is similarly effective in the case of the nanosystems obtained through microwave-assisted hydrothermal method, which is in accordance with the TG-DSC results which show a similar loading for the two samples, i.e., 6.32% and 6.67%, respectively.

## 3. Discussion

Nanomaterial-based drug delivery systems have gained a great amount of research interest due to their ability to improve drug stability and water solubility, prolong blood circulation time, and ensure targeted drug release and uptake [2,31,32]. In this context, this study aimed to develop two types of MNP-based drug delivery systems for the delivery of UA that could potentially be applied as anticancer systems.

The starting point within the design of the study involves the need for obtaining nanostructures with uniform properties in terms of size, shape, surface chemistry, and structure, which could mainly be achieved in the synthesis step. The most common synthesis technique for MNPs involves the co-precipitation of the iron ions into an alkaline environment. Although it is the preferred route owing to its simplicity and economic feasibility, co-precipitation leads to significant variations in the properties of the nanoparticles, especially regarding the broad size distributions [26,33]. Therefore, this study focused on comparing the outcome properties of UA-loaded MNPs obtained through two synthesis methods, namely the co-precipitation and microwave-assisted hydrothermal method.

As it was demonstrated within this study, the microwave-assisted hydrothermal method significantly limited the variance between the samples, allowing for a more controlled and uniform drug loading. Specifically, while the size of the MNPs is larger when compared to the ones obtained through co-precipitation, the size distributions are significantly narrower, especially for the UA-loaded samples. The increase in nanoparticle size is consistent with previously available studies [26,34,35], which is caused by the application of high pressure and temperature conditions. Nonetheless, the use of larger nanoparticles in biomedical applications is generally preferred, as they allow for preventing agglomeration and accumulation, while simultaneously avoiding rapid body clearance [36,37]. Moreover, obtaining MNPs with slightly increased diameters further improves their magnetic properties, which could be of great benefit in hyperthermia applications. In this context, the present study successfully demonstrated the potential of the microwave-assisted hydrothermal method to obtain superparamagnetic iron oxide nanoparticles with higher saturation magnetization, i.e., between 50 and 70 emu/g, without the need for additional coatings as it was previously reported in the literature [21,38].

Another important aspect of the study is represented by the use of a natural biocompound as a potential anticancer agent that would significantly limit the side effects of conventional chemotherapeutics. UA is one of the four main lichen secondary metabolites with a plethora of biomedical and pharmaceutical properties, including antimicrobial, antitumor, antioxidant, analgesic, anti-inflammatory, and UV-protective effects [13,39]. While studies generally focused on UA efficacy against a wide spectrum of microbial species, i.e., Gram-positive and Gram-negative bacteria [40,41,42,43,44,45,46], viruses [47,48,49,50], fungi [51,52,53], and protozoa [54], recent trends have shifted towards its use in anticancer therapy due to demonstrated antiproliferative and cytotoxic effects against numerous cancer cell lines [39,55]. In this context, literature reports numerous studies focusing on the application of UA in cancer treatment using both nano- and microscaled drug delivery systems, such as liposomes, nanoemulsions, polymeric nanocapsules, nanospheres, nanofibers, microspheres, nanodiamonds, and magnetic nanoparticles [56]. While most of the magnetic nanoparticle-based studies focused on their antimicrobial activity, Alpsoy et al. reported an increased antitumoral activity of MNPs functionalized with (3-aminopropyl)triethoxysilane, followed by the surface conjugation of carboxylated polyethylene glycol, folic acid, and UA against the L929 and A549 cancer cells as compared to other cancer cells, i.e., U87, HeLa, and MCF-7 [19].

Therefore, the anticancer activity of the UA-loaded MNPs reported within this study represents a step forward in the development of nanostructured drug delivery systems suitable for hyperthermia applications.

## 4. Materials and Methods

### 4.1. Materials

Ferric chloride hexahydrate (FeCl_3_·6H_2_O), ferrous sulphate heptahydrate (FeSO_4_·7H_2_O), sodium hydroxide (NaOH), and UA were purchased from Sigma–Aldrich Merck (Darmstadt, Germany) and were used without additional purification.

The HEK 293T cell line was provided by the National Research Center for Micro and Nanomaterials, University Politehnica of Bucharest.

### 4.2. Nanoparticle Synthesis

Similar to the procedures described in our previous study [26], two different methods were employed for the synthesis of MNPs, namely the co-precipitation and microwave-assisted hydrothermal method. For the co-precipitation method, FeCl_3_·6H_2_O and FeSO_4_·7H_2_O were dissolved in 350 mL of distilled water in a 2:1 molar ratio at the final concentration of 1%; using a peristaltic pump, the solution was dripped at 100 rpm into 150 mL of 1M NH_4_OH solution. For the UA-loaded samples, UA was dissolved in the NH_4_OH solution at three different mass concentrations, namely 5, 10, and 15%. The resulting precipitate was decanted using a NdFeB magnet and washed with distilled water until a neutral pH. For the microwave-assisted hydrothermal method, the precipitate was transferred to a Teflon vial of 800 mL capacity and further introduced into the Milestone Synthwave equipment. The reaction parameters included 60 bar (N_2_) pressure, 80 °C temperature, 30 min reaction time, and 10% stirring. Similarly, the so-obtained nanoparticles were washed with distilled water until a neutral pH and dried overnight at 40 °C. Samples were stored at room temperature. Table 4 summarizes the samples obtained and their coding, which will be used throughout the manuscript.

### 4.3. Morpho-Structural and Physicochemical Characterization

#### 4.3.1. X-ray Diffraction (XRD)

The XRD analysis was performed on a CuKα radiation equipped PANalytical Empyrean diffractometer (PANalytical, Almelo, The Netherlands). Measurements were carried out within the 10–80 ° 2θ angle range, at the step size of 0.0256 ° and time per step of 1 s. The obtained diffractograms were fitted using the Rietveld refinement algorithm within the HighScore Plus software (version 3.0, PANalytical, Almelo, The Netherlands). Goodness of fit < 4 was considered acceptable for diffractogram fittings.

#### 4.3.2. Fourier Transform Infrared Spectroscopy (FT-IR)

A Thermo Scientific Nicolet iS50 (Thermo Fischer Scientific, Waltham, MA, USA) spectrometer equipped with a liquid nitrogen-cooled mercury cadmium telluride detector was used for the acquisition of IR spectra. The measurement parameters involved the attenuated total reflectance (ATR) mode, room temperature, 4 cm^−1^ resolution, and the range of 4000–400 cm^−1^. The OmnicPicta software (version 8.2, Thermo Nicolet, Thermo Fischer Scientific, Waltham, MA, USA) was used for the co-addition and processing of the 64 scans performed on each sample.

#### 4.3.3. Thermogravimetry and Differential Scanning Calorimetry (TG-DSC)

The UA loading was estimated on all samples by subjecting them to the TG-DSC analysis on an STA TG/DSC Netzsch Jupiter 449 F3 equipment (Selb, Germany). Samples were introduced within an alumina crucible and the thermal treatment involved a heating rate of 10 K/min in the range of 20–900 °C in a dynamic air atmosphere of 50 mL/min.

#### 4.3.4. Dynamic Light Scattering (DLS) and Zeta Potential

For the DLS and zeta potential measurements, 5 mg of each sample was dispersed in 15 mL of PBS 1x solution (7.4 pH) for 10 min at room temperature using a Sonorex Digitec DT 514 ultrasonic bath (Bandelin, Berlin, Germany) with an ultrasonic peak power of 860 W. The suspension was placed inside the measurement cell and introduced into the DelsaMax Pro equipment (Backman Coulter, Brea, CA, USA). Triplicate measurements were carried out for each sample.

#### 4.3.5. Scanning Electron Microscopy (SEM)

A small amount of each sample was fixated onto a carbon band and introduced into the Inspect F50 high-resolution microscope (Thermo Fisher—former FEI, Eindhoven, The Netherlands). Micrographs at different magnifications were obtained using secondary electrons at the energy of 30 KeV and beam spot of 5.5.

Based on the obtained micrographs, 100 nanoparticles were measured using the ImageJ software (https://imagej.nih.gov/ij/download/) in order to determine the average nanoparticle size and the size distribution for each type of drug delivery system.

#### 4.3.6. Vibrating Sample Magnetometry (VSM)

The magnetic properties of the UA-loaded MNPs were investigated through the VSM analysis (VSM, VersaLabTM 3T, Cryogen-free Vibrating Sample Magnetometer, Westerville, OH, USA). The magnetic field applied ranged between −10 and +10 kOe and vice versa, with a step rate of 10 Oe/s, at room temperature (25 °C).

### 4.4. Biological Evaluation

The XTT assay was conducted on a HEK 293T cell line. Initially, all samples were sterilized for 1 h using UV light. Each sample was tested at the concentration of 10 mg/mL by dispersing 2 mg of each sample into 200 µL cell suspension for 24, 48, and 72 h exposure. After incubation, 70 μL of the freshly prepared working solution containing the XTT reagent (2,3-Bis-(2-Methoxy-4-Nitro-5-Sulfophenyl)-2H-Tetrazolium-5-Carboxanilide) and an electron coupling reagent was added and incubated for 4 h at 37 °C and 5% CO_2_. The optical density (OD) was spectrophotometrically determined at 450 nm using a SpectraMax i3x Multi-Mode microplate reader and the SoftMax Pro 6 software. The cell viability of the samples was calculated using the following equation:

(1) $$\mathrm{Cell}\;\mathrm{viability}\left(\%\right)=\frac{\mathrm{OD}450\;\mathrm{sample}}{\mathrm{OD}450\;\mathrm{control}}\;\times \;100$$

Statistical evaluation was performed using the Student’s *t*-test, for which the significance levels were considered as * *p* < 0.05, ** *p* < 0.01, and *** *p* < 0.001.

## 5. Conclusions

The starting point within the design of the present study was based upon the need to achieve more uniform outcome properties of the drug delivery systems through the synthesis method. In this context, two types of synthesis methods were compared in regard to the drug loading efficiency, size, stability, and magnetic properties of the UA-loaded MNPs. Results demonstrated that the use of the microwave-assisted hydrothermal method leads to slightly increased nanoparticle diameters with considerably narrower size distributions, increased drug loading efficiencies, lower agglomeration tendencies, and higher saturation magnetization values. Thus, in comparison to the co-precipitation synthesis, the microwave-assisted hydrothermal method offers significant advantages regarding the obtaining of drug nanocarriers for the targeted delivery of biocompounds. Moreover, the use of UA proved to be efficient against the HEK 293T cell line as seen through the XTT assay, thus demonstrating the potential of the developed nanostructures to be further applied in cancer treatment.

## Figures and Tables

**Table 1:** Unit cell parameters, average crystallite size, and crystallinity acquired from the Rietveld refinement of the diffractograms.

Sample	Unit Cell Parameters		Average Crystallite Size ± Standard Deviation (SD) [nm]
	a = b = c [Å]	α = β = γ [°]	
MNP_CP	8.36	90	8.13 ± 0.17
MNP@5UA_CP	8.36	90	7.66 ± 0.19
MNP@10UA_CP	8.35	90	5.80 ± 0.23
MNP@15UA_CP	8.34	90	5.33 ± 0.26
MNP_HT	8.35	90	12.42 ± 0.80
MNP@5UA_HT	8.35	90	10.88 ± 0.46
MNP@10UA_HT	8.35	90	9.20 ± 0.59
MNP@15UA_HT	8.35	90	9.45 ± 0.31

**Table 2:** The thermal effects, mass loss, and estimated UA loading for the pristine and UA-loaded MNPs.

Sample	Mass Loss RT-200 °C (%)	Endothermic Effect (°C)	Mass Loss 200–400 °C (%)	Exothermic Effect (°C)	Residual Mass (%)	Estimated Load (%)
MNP_CP	3.20	77.1	1.70	592.2	94.44	-
MNP_5UA_CP	3.54	61.9	1.95	601.1	93.89	0.58
MNP_10UA_CP	4.10	67.0	5.18	574.4	89.79	4.92
MNP_15UA_CP	3.84	86.4	6.79	577.1	88.47	6.32
MNP_HT	1.92	63.1	1.37	617.8	95.94	-
MNP_5UA_HT	1.82	75.4	3.69	613.2	93.61	2.43
MNP_10UA_HT	2.07	82.5	7.37	598.8	89.54	6.67
MNP_15UA_HT	2.18	84.4	9.13	591.6	87.56	8.73

**Table 3:** The saturation magnetization (Ms), remanence magnetization (Mr), and coercivity field (Hc) values of the pristine and UA-loaded MNPs.

Sample	Ms (emu/g)	Mr (emu/g)	Hc (Oe)
MNP_CP	59.55	0.54	4.97
MNP@5UA_CP	59.14	0.53	4.26
MNP@10UA_CP	47.79	0.86	11.02
MNP@15UA_CP	46.12	0.71	9.22
MNP_HT	72.05	2.97	19.09
MNP@5UA_HT	68.08	1.60	13.80
MNP@10UA_HT	62.37	1.23	11.76
MNP@15UA_HT	52.20	0.65	7.04

**Table 4:** Summary of the UA-loaded MNP samples obtained.

Sample	Synthesis Method	UA Concentration (wt. %)
MNP_CP	co-precipitation	0
MNP@5UA_CP		5
MNP@10UA_CP		10
MNP@15UA_CP		15
MNP_HT	microwave-assisted hydrothermal method	0
MNP@5UA_HT		5
MNP@10UA_HT		10
MNP@15UA_HT		15

## Supplementary Materials

The following supporting information can be downloaded at: https://www.mdpi.com/article/10.3390/molecules28135198/s1, Figure S1: Hydrodynamic diameter distribution for sample MNP_CP; Figure S2: Hydrodynamic diameter distribution for sample MNP@5UA_CP; Figure S3: Hydrodynamic diameter distribution for sample MNP@10UA_CP; Figure S4: Hydrodynamic diameter distribution for scample MNP@15UA_CP; Figure S5: Hydrodynamic diameter distribution for sample MNP_HT; Figure S6: Hydrodynamic diameter distribution for sample MNP@5UA_HT; Figure S7: Hydrodynamic diameter distribution for sample MNP@10UA_HT; Figure S8: Hydrodynamic diameter distribution for sample MNP@15UA_HT.
