# Supplementary material for: Usnic Acid-Loaded Magnetite Nanoparticles—A Comparative Study between Synthesis Methods
Source: Molecules. 2023 Jul 4;28(13):5198. doi: 10.3390/molecules28135198 (PMC10343309; doi:10.3390/molecules28135198)
Supplement: Supplementary file 1 [file molecules-28-05198-s001.zip › molecules-2420892-supplementary.pdf]

Usnic Acid-Loaded Magnetite Nanoparticles – A Comparative Study Between Synthesis Methods

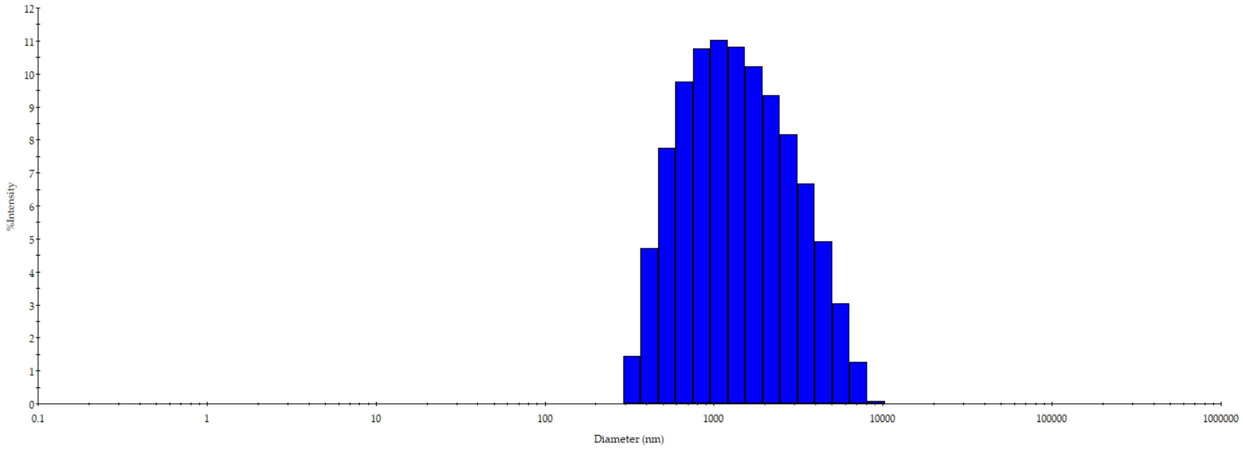

Figure S1: Hydrodynamic diameter distribution for sample MNP_CP.

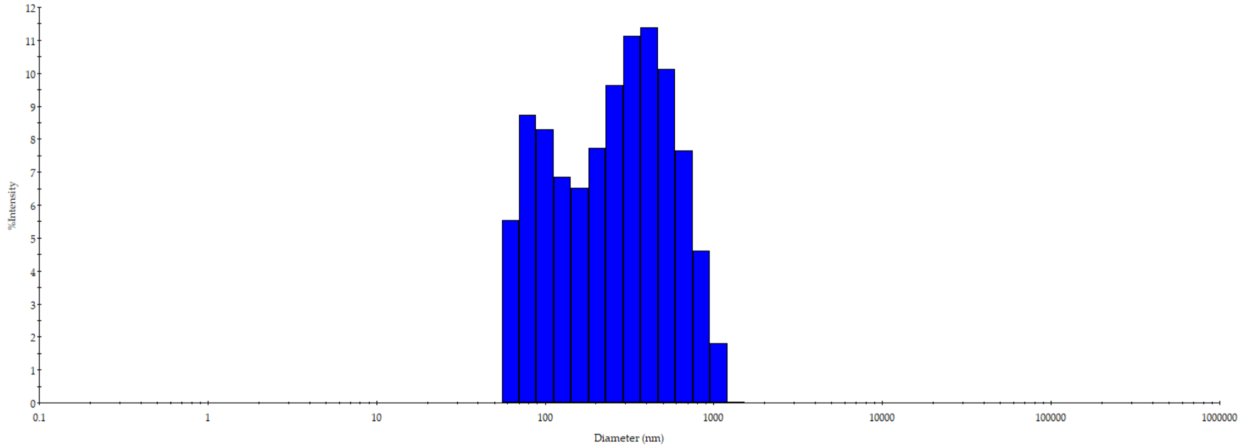

Figure S2: Hydrodynamic diameter distribution for sample MNP@5UA_CP.

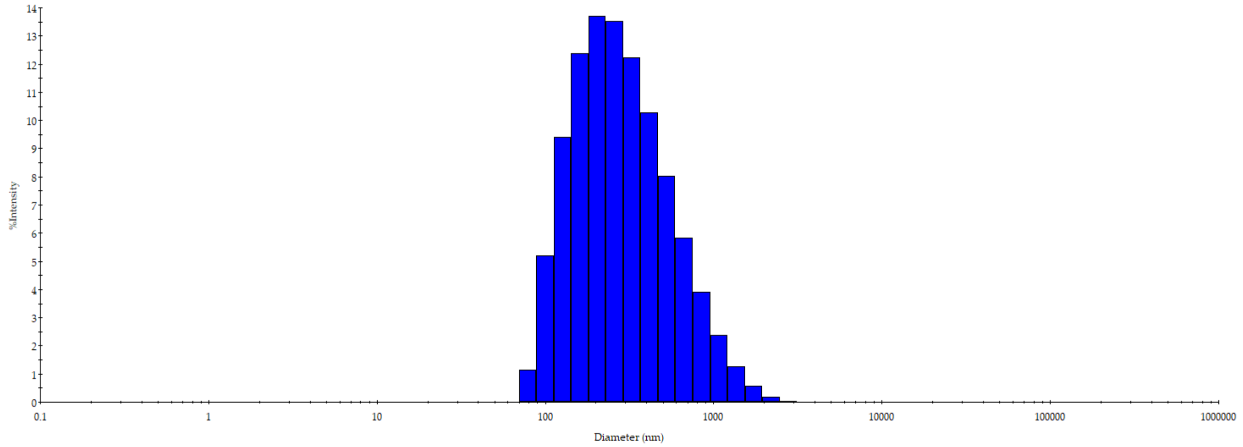

Figure S3: Hydrodynamic diameter distribution for sample MNP@10UA_CP.

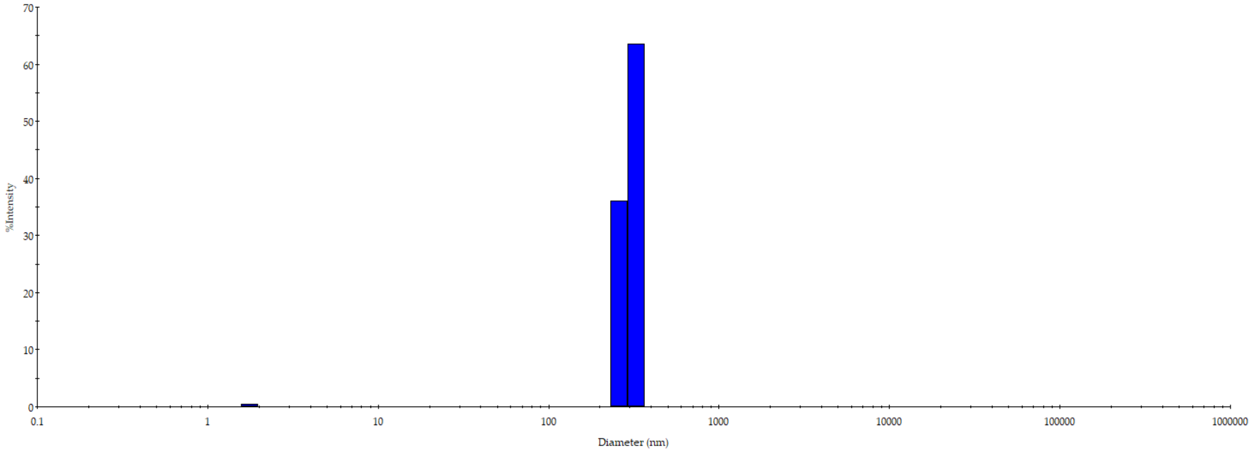

Figure S4: Hydrodynamic diameter distribution for sample MNP@15UA_CP.

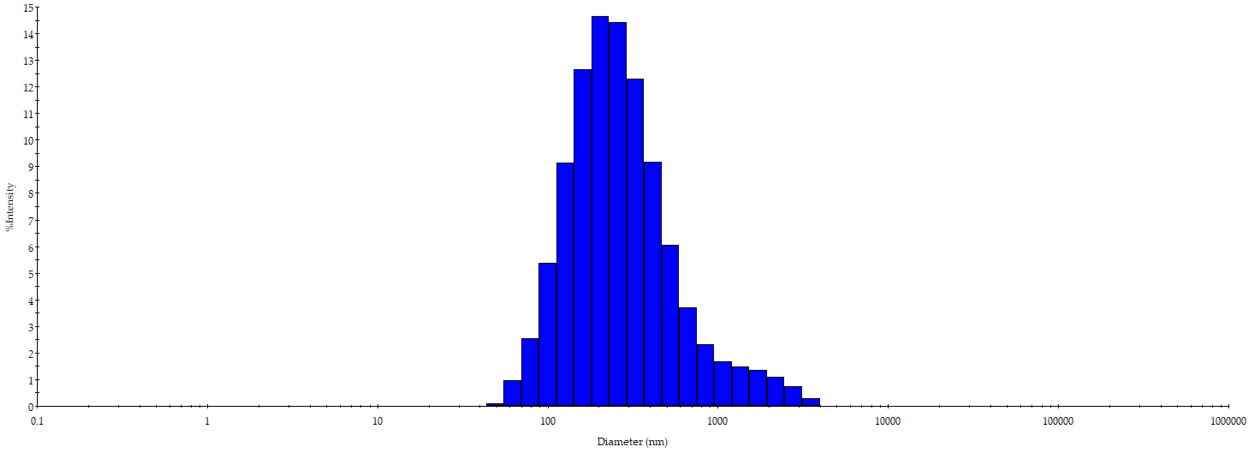

Figure S5: Hydrodynamic diameter distribution for sample MNP_SW.

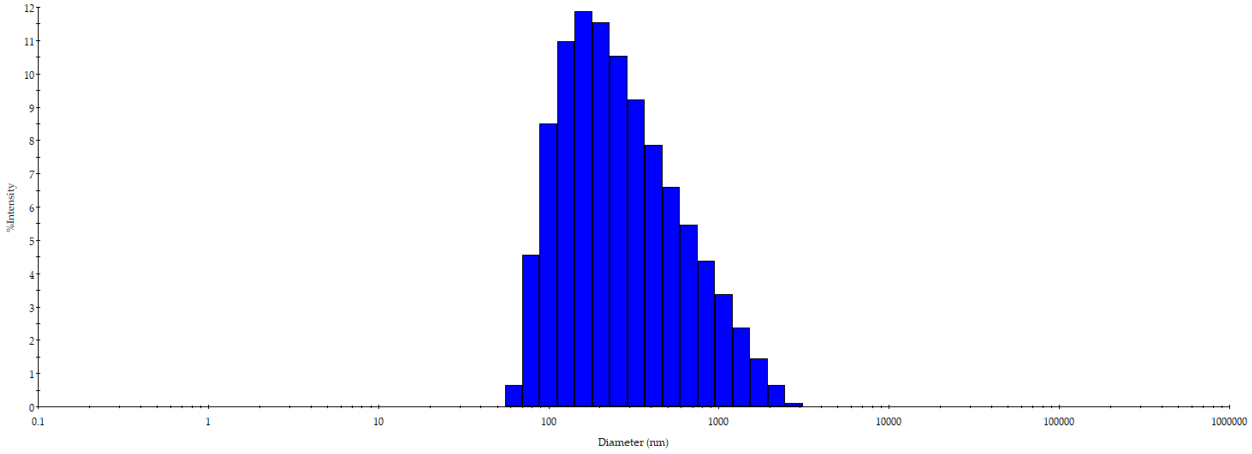

Figure S6: Hydrodynamic diameter distribution for sample MNP@5UA_SW.

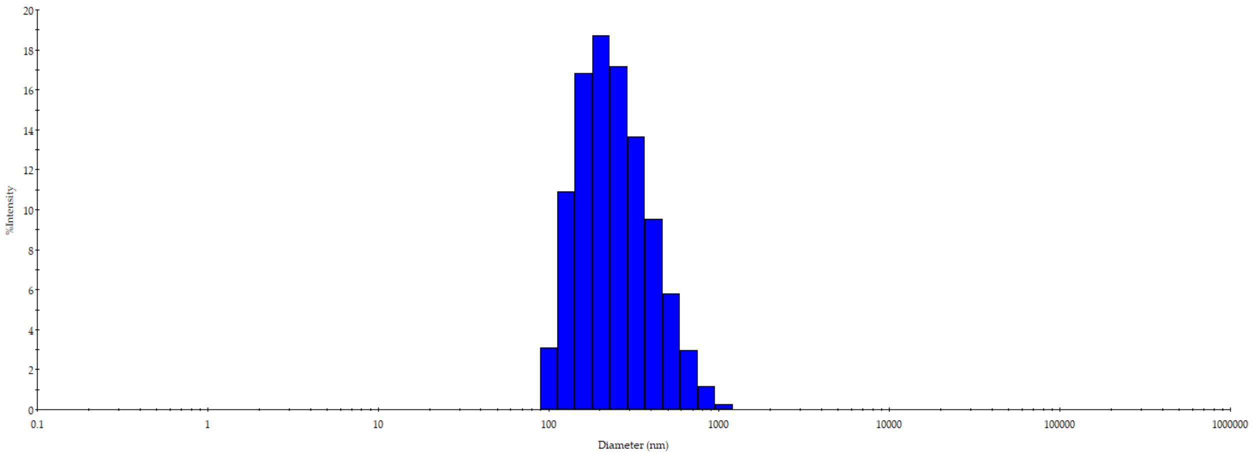

Figure S7: Hydrodynamic diameter distribution for sample MNP@10UA_SW.

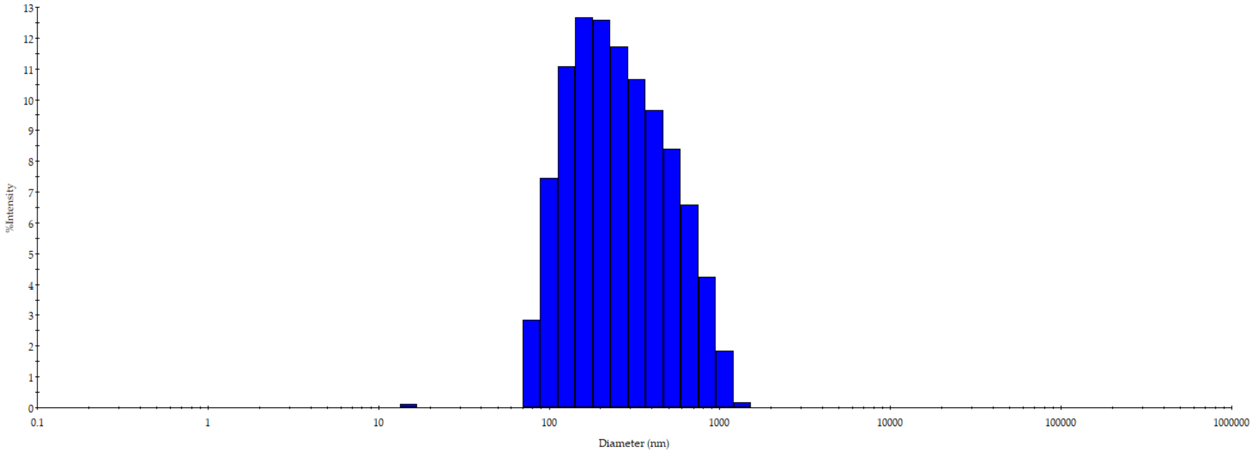

Figure S8: Hydrodynamic diameter distribution for sample MNP@15UA_SW.
